# Risk Factors for Early Implant Failure Following Sinus Augmentation: A Multi‐Centre Nested Case–Control Study

**DOI:** 10.1111/jcpe.14191

**Published:** 2025-06-05

**Authors:** Björn Bonsmann, Mohamed Abughalia, Constantin von See, Thomas Dietrich

**Affiliations:** ^1^ Danube Private University Krems Austria; ^2^ Department of Oral Surgery, Faculty of Dentistry Sirte University Sirte Libya; ^3^ Department of Digital Technologies in Dentistry and CAD/CAM Danube Private University Krems Austria; ^4^ The School of Dentistry University of Birmingham Birmingham UK; ^5^ Department of Oral Surgery, Birmingham Dental Hospital Birmingham Community Healthcare NHS Foundation Trust Birmingham UK; ^6^ NIHR Birmingham Biomedical Research Centre University of Birmingham Birmingham UK

**Keywords:** early implant failure, periodontitis, risk factors, sinus lift

## Abstract

**Aim:**

To evaluate the determinants of early implant failure after sinus augmentation (SA).

**Materials and Methods:**

We conducted a nested case–control study of implants placed after SA between 2016 and 2021 in eight centres across Germany. We included a total of 129 implants that were lost within 12 months after placement (cases) and 273 implants that were not lost within 12 months (controls). Multivariable logistic regression models were fitted to estimate odds ratios (ORs) and 95% confidence intervals for the association of various clinical and radiographic parameters with the risk of early implant failure, independent of confounders.

**Results:**

Marked associations with early failure risk were found for male sex (OR 1.6, 95% CI: 1.1–2.5), current smoking (OR 1.9, 95% CI: 1.0–3.6), history of severe periodontitis (OR 7.5, 95% CI: 2.4–23), number of missing teeth (OR 1.4, 95% CI: 1.2–1.6), residual bone height of 3 to < 5 mm (OR 2.4, 95% CI: 1.4–4.2) and < 3 mm (OR 3.8, 95% CI: 2.2–6.8) and insertion torque < 8 Ncm (OR 5.8, 95% CI 1.3–26).

**Conclusion:**

Male sex, current smoking, low residual bone height, very low insertion torque, a history of severe periodontitis and number of missing teeth are associated with early implant failure after SA.

## 1 Introduction

Implant placement in the posterior maxilla can be a challenge in many cases because of the lack of bone due to bone loss following tooth extraction and progressive pneumatisation of the maxillary sinus. An additional complicating factor is the poorer bone quality in the posterior maxilla when compared to other regions of the alveolar process (Sogo et al. 2012).

Maxillary sinus floor augmentation (sinus lift) is a common procedure to address the lack of bone height to enable placement of dental implants of the desired length. External (lateral approach) maxillary sinus augmentation (SA) was first performed over 50 years ago by Hill Tatum and described by Boyne and James (1980). For cases with sufficient residual bone height, an alternative internal (transcrestal) approach was suggested later (Summers 1994).

Both internal and external maxillary SA are predictable treatments associated with high success rates in terms of implant survival (Pjetursson et al. 2008; Raghoebar et al. 2019). Five‐year survival reported in the literature ranges between 88% and 100% of implants (Raghoebar et al. 2019). However, implant failures do happen, and most (i.e., four out of five) implant failures occur within the first year after placement (Del Fabbro et al. 2013).

Identifying patient‐ or site‐related risk factors and/or procedural determinants of implant failure in the context of maxillary SA is extremely challenging given the large sample sizes required for evaluating what is a relatively rare outcome. Therefore, evidence for risk factors of implant failure in the context of SA is scarce, although male sex, smoking, lower residual bone height and the surgeon's experience have been reported to be associated with implant failure (Li et al. 2023; Testori et al. 2012).

The aim of the present study was to identify the risk factors for early implant failure within 12 months of placement in the context of sinus floor augmentation using data from a large multi‐centre cohort.

## 2 Materials and Methods

### 2.1 Data Source

The European Centres for Dental Implantology (ECDI) are an association of oral and maxillofacial/oral surgeons working in private practices with a significant implantology portfolio (hereafter referred to as centres). Almost all centres are located in Germany and work as referral practices: that is, patients are referred by their general dentists for implant surgery. For almost all patients, implant restorations will be provided by the referring dentist. Since 2010, ECDI members have documented their implant surgeries for quality control purposes using a commercially available implant documentation software (impDAT, Kea Software GmbH, Tutzing, Germany). In 2016, data collection was modified to include more detailed data on augmentations including sinus lift procedures. Patient‐, procedure‐ and implant‐related data were recorded at the time of surgery and, where applicable, subsequent appointments (e.g., second‐stage surgery/abutment connection) or follow‐up appointments due to complications.

A total of 90,700 implants in 39,392 patients placed in 20 centres were recorded in the database from 2016 to 2021. In connection with sinus floor elevations, 18,665 implants were documented, of which 281 implants in 231 patients were recorded as lost within 1 year after placement (early implant failures).

In order to identify determinants of early failure of implants placed in the context of sinus lift procedures, we conducted a nested case–control study using the ECDI database as the study base. Cases were defined as implants placed in the context of a sinus lift surgery, irrespective of staging (one‐stage/two‐stage) or approach (transcrestal/lateral window), that had been lost within 12 months after placement. Because of resource limitations, the study was restricted to eight centres, which had contributed a total of 167 cases to the database. For each centre, a random list was generated of implants placed in the context of sinus lift procedures that had not been recorded as lost within 12 months after placement (controls). We planned to include as many controls as cases from each centre, but aimed to include more controls where possible to increase statistical power subject to available resources.

The following parameters were extracted from the database: patient age at implant placement, sex, smoking history (never, former, current), implant site, implant length and diameter, type of sinus lift (transcrestal/lateral window), staging of sinus lift surgery (one‐stage vs. two‐stage), type of implant healing (submerged vs. transmucosal), perforation of the Schneiderian membrane during sinus lift (yes/no) and insertion torque (20+ Ncm, 8–19 Ncm, < 8 Ncm).

For each included case and control implant, post‐operative panoramic radiographs were retrieved from the medical records, and the following assessments were made by the first author: maximum periodontal bone loss (as a percentage of root length) on the worst affected tooth, residual bone height prior to sinus graft (in mm) and bone height after sinus graft (in mm) and the number of remaining teeth (not including third molars). Radiographic measurements were performed using the open‐source software ImageJ (National Institutes of Health, Bethesda, USA) (Schneider et al. 2012). The known implant length was used as a reference to calibrate measurements obtained from panoramic radiographs. Measurements were repeated on 32 radiographs, which showed excellent reliability for all radiographic measurements (intraclass correlation coefficients > 0.98).

Study data were collected and managed using REDCap electronic data capture tools hosted at the University of Birmingham (Harris et al. 2019, 2009).

Of the 167 cases in the ECDI database, we excluded 22 cases for whom no records/radiographs could be retrieved and a further 4 cases where radiographs were deemed of insufficient quality. Of the remaining 141 cases, 9 patients had two failed implants each and 1 patient had four failed implants. For these patients, one implant was randomly chosen for inclusion, resulting in a final sample of 129 case implants. Of the 331 randomly selected controls from the database, 32 were excluded because of missing records/radiographs and a further 13 were excluded for insufficient quality radiographs. Of the remaining 286 controls, 13 patients had two control implants each, one of which was chosen at random for inclusion. Thus, a total of 273 randomly selected control implants were included in the final sample.

### 2.2 Statistical Analysis

Summary statistics for study parameters were calculated as appropriate by case/control status. To evaluate determinants of early failure, multivariable logistic regression models were fitted with case/control status as the dependent variable, adjusting for centre. Directed acyclic graphs specifying causal hypotheses were constructed to inform which variables were used in multivariable models to adjust for confounding when evaluating the association between the specific exposure variables of interest and the risk of early implant failure. This was done to ensure that no variables in the causal pathway were inappropriately included in a model (Hernán and Robins 2020). In addition, we omitted potential confounders using a 10% change‐in‐estimate criterion to reduce the number of parameters in the final model; however, age and sex were retained in all models. Residual bone height and maximum periodontal bone loss were modelled both as continuous variables and in categories to enhance interpretability. We also calculated the ratio of maximum bone loss and age as a measure of periodontitis grade according to the 2018 classification (Papapanou et al. 2018). We tested for non‐linearity of the association with early implant failure for periodontal bone loss, bone loss/age ratio, number of missing teeth and residual bone height by adding quadratic terms and using likelihood ratio tests at *α* = 0.1. We also explored the association between residual bone height and periodontal bone loss as well as between residual bone height and primary stability using linear regression, and between smoking status and perforations of the Schneiderian membrane using logistic regression. Missing data on smoking history were coded as missing; otherwise, all analyses were complete case analyses. Results are reported as odds ratios (ORs) with two‐sided 95% confidence intervals. Model fit of all models was confirmed with the Hosmer–Lemeshow goodness‐of‐fit test. All analyses were performed with STATA 18.5 (StataCorp, College Station, TX, USA).

The study protocol was reviewed by the Ethics Committee of Danube Private University (GZ: DPU‐EK/040). This manuscript was prepared in accordance with STROBE guidelines (https://www.strobe‐statement.org/).

## 3 Results

The final analysis sample included 402 patients and implants (129 cases and 273 controls). Patient, implant and procedural characteristics of the study sample are presented in Table 1. The mean age of the patients was 60 years (range: 26–88 years); 55% were male and 50% were never smokers. Thirty‐nine percent had evidence of periodontal bone loss on panoramic radiographs. The majority of implants were placed at the time of the sinus lift (one‐stage procedure), and most implants were placed in the first molar region. Approximately three out of four implants were left to heal submerged (Table 1).

**TABLE 1:** Summary statistics for study parameters by case/control status.

	Controls	Cases	Total
*N*	273	129	402
Centre, *n* (%)			
1	68 (24.9%)	29 (22.5%)	97 (24.1%)
2	46 (16.8%)	21 (16.3%)	67 (16.7%)
3	24 (8.8%)	16 (12.4%)	40 (10.0%)
4	34 (12.5%)	17 (13.2%)	51 (12.7%)
5	15 (5.5%)	10 (7.8%)	25 (6.2%)
6	34 (12.5%)	18 (14.0%)	52 (12.9%)
7	18 (6.6%)	8 (6.2%)	26 (6.5%)
8	34 (12.5%)	10 (7.8%)	44 (10.9%)
Age (years), mean (SD)	60.0 (11.2)	60.4 (9.9)	60.1 (10.8)
Sex, *n* (%)			
Female	135 (49.5%)	48 (37.2%)	183 (45.5%)
Male	138 (50.5%)	81 (62.8%)	219 (54.5%)
Regular medication intake, *n* (%)	132 (56.2%)	61 (57.0%)	193 (56.4%)
Smoking status, *n* (%)			
Never	117 (51.1%)	50 (47.2%)	167 (49.9%)
Former	73 (31.9%)	28 (26.4%)	101 (30.1%)
Current	39 (17.0%)	28 (26.4%)	67 (20.0%)
Number of teeth, mean (SD)	18.0 (6.9)	13.4 (8.1)	16.5 (7.6)
History of periodontitis, *n* (%)			
No bone loss	157 (57.5%)	57 (44.2%)	214 (53.2%)
BL coronal third	56 (20.5%)	17 (13.2%)	73 (18.2%)
BL middle third	41 (15.0%)	26 (20.2%)	67 (16.7%)
BL apical third	5 (1.8%)	13 (10.1%)	18 (4.5%)
Edentulism	14 (5.1%)	16 (12.4%)	30 (7.5%)
Ratio bone loss (%)/age (years), *n* (%)			
No bone loss	157 (57.5%)	57 (44.2%)	214 (53.2%)
< 0.25	0 (0%)	0 (0%)	0 (0%)
0.25–1.0	92 (33.7%)	43 (33.3%)	135 (33.6%)
> 1.0	10 (3.7%)	13 (10.1%)	23 (5.7%)
Edentulism	14 (5.1%)	16 (12.4%)	30 (7.5%)
Staging of sinus lift, *n* (%)			
One‐stage	221 (81.0%)	88 (68.2%)	309 (76.9%)
Two‐stage	52 (19.0%)	41 (31.8%)	93 (23.1%)
Type of sinus lift, *n* (%)			
Internal	30 (11.0%)	10 (7.8%)	40 (10.0%)
External	242 (89.0%)	119 (92.2%)	361 (90.0%)
Site, *n* (%)			
First premolar	33 (12.1%)	6 (4.7%)	39 (9.7%)
Second premolar	55 (20.1%)	30 (23.3%)	85 (21.1%)
First molar	139 (50.9%)	80 (62.0%)	219 (54.5%)
Second molar	46 (16.8%)	13 (10.1%)	59 (14.7%)
Healing mode			
Transmucosal	72 (27.0%)	35 (27.6%)	107 (27.2%)
Submerged	195 (73.0%)	92 (72.4%)	287 (72.8%)
Residual bone height (mm), mean (SD)	5.7 (2.4)	4.3 (2.8)	5.3 (2.6)
Bone height with graft (mm), mean (SD)	12.0 (2.0)	11.3 (2.2)	11.7 (2.1)
Implant diameter (mm), mean (SD)	4.2 (0.5)	4.2 (0.4)	4.2 (0.5)
Implant length (mm), mean (SD)	10.6 (1.2)	10.3 (1.4)	10.5 (1.3)
Insertion torque, *n* (%)			
20+ Ncm	209 (77.4%)	93 (74.4%)	302 (76.5%)
8–19 Ncm	57 (21.1%)	23 (18.4%)	80 (20.3%)
< 8 Ncm	4 (1.5%)	9 (7.2%)	13 (3.3%)

**FIGURE 1:**
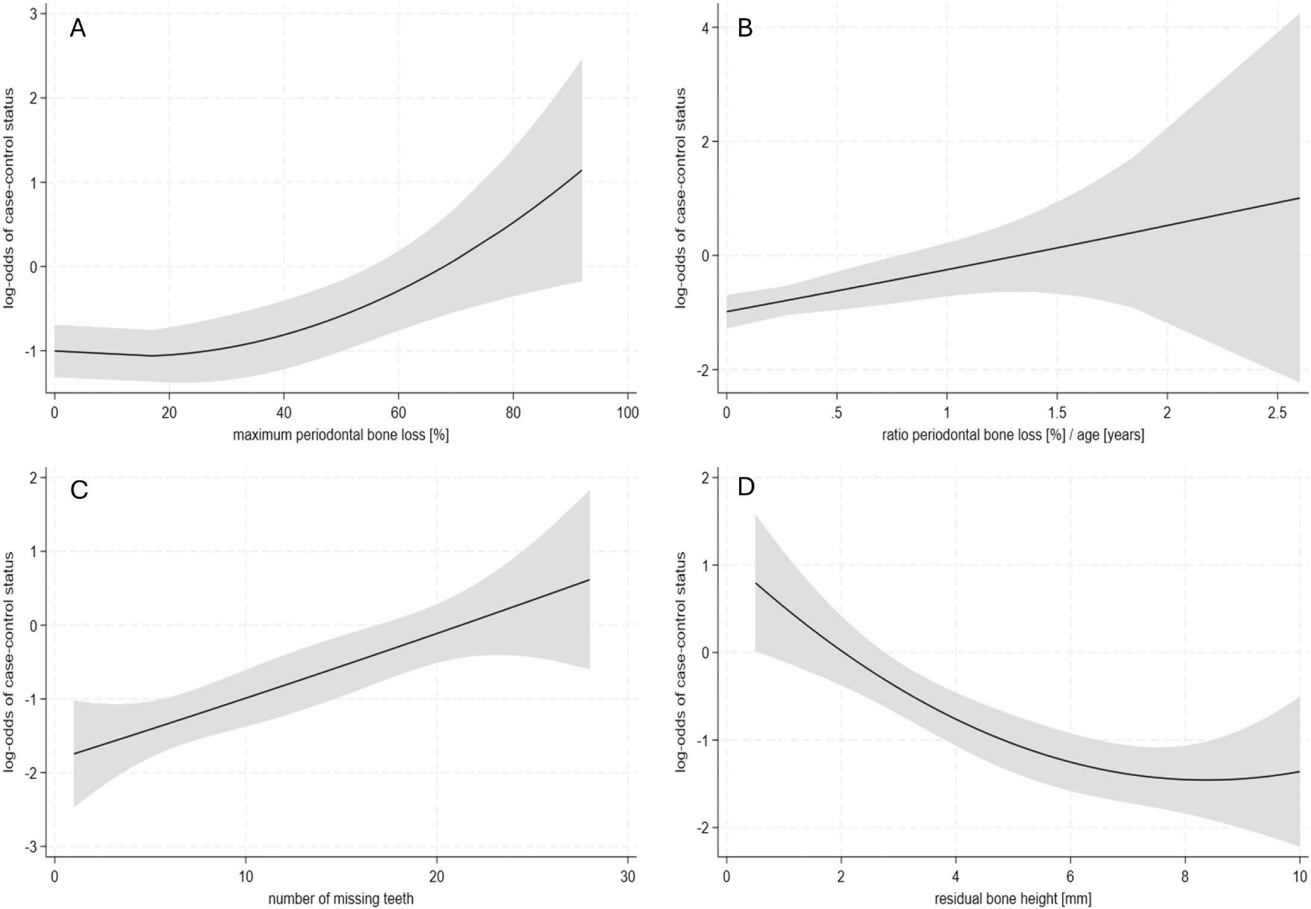
Association between maximum periodontal bone loss (panel A, *p* = 0.003, *p* for non‐linearity = 0.05), maximum periodontal bone loss/age ratio (panel B, *p* = 0.009, *p* for non‐linearity = 0.88), number of missing teeth (panel C, *p* < 0.0001, *p* for non‐linearity = 0.95, excluding third molars), residual bone height (panel D, *p* < 0.0001, *p* for non‐linearity = 0.06) and risk of early implant failure.

**FIGURE 2:**
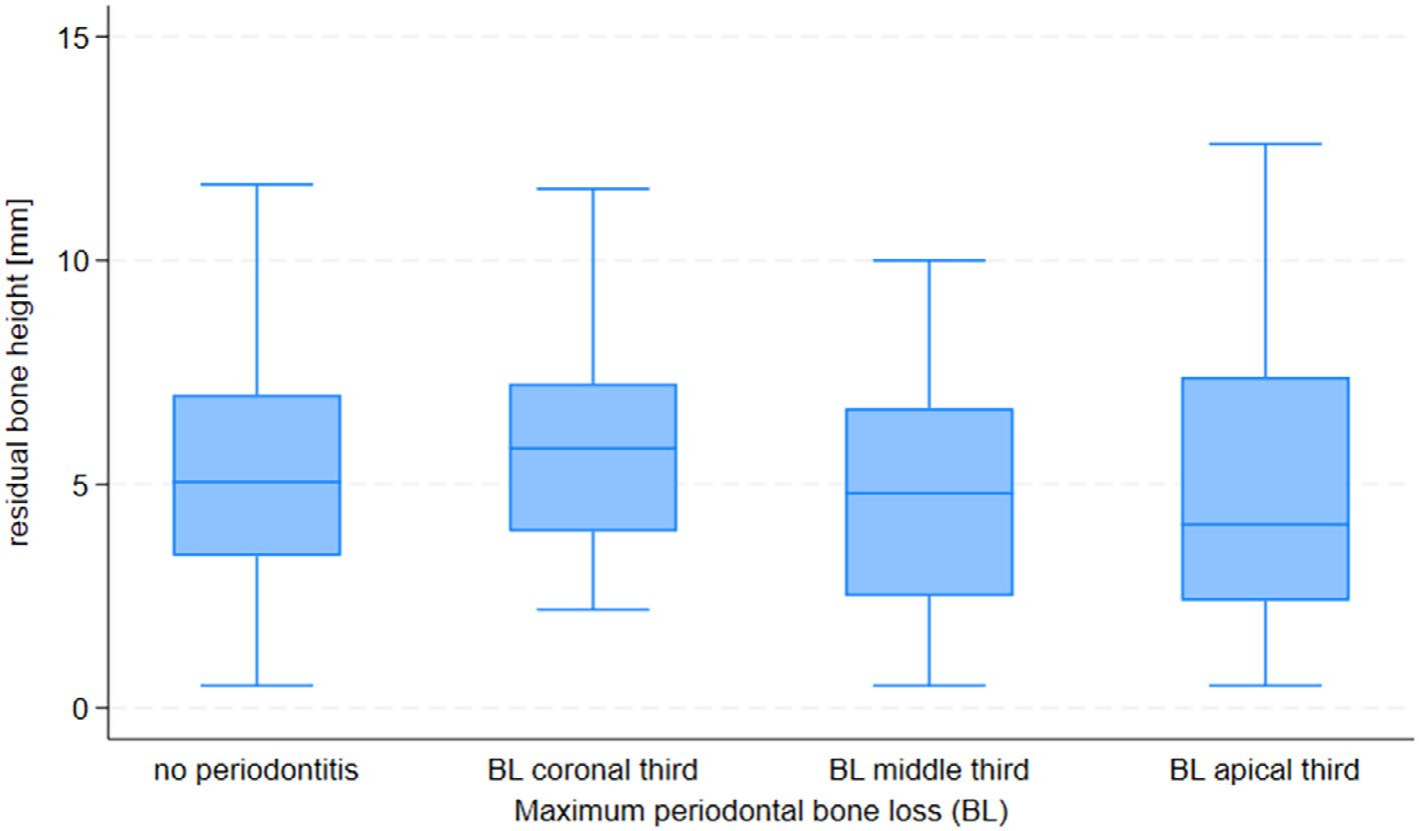
Box plot showing residual bone height before sinus lift surgery (in mm) by category of maximum periodontal bone loss (BL), *p* for trend = 0.364.

**FIGURE 3:**
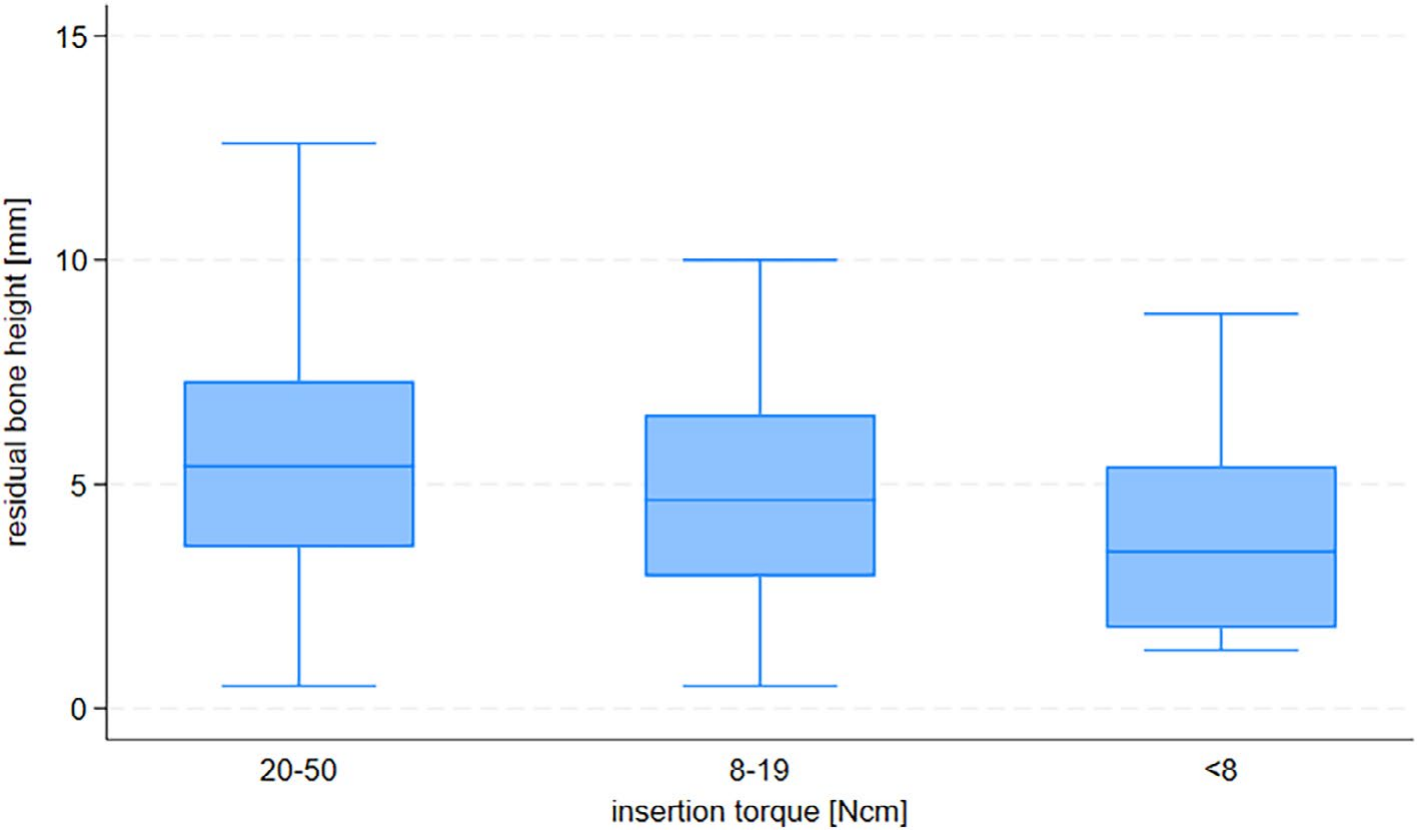
Box plot showing residual bone height before sinus lift surgery (in mm) by category of insertion torque (in N cm), *p* for trend = 0.005.

Multivariable logistic regression showed that age was not associated with an increased risk of early failure (Table 2). However, men were 60% more likely than women to experience early implant failure (OR 1.6, 95% CI: 1.1–2.5). Compared to never smokers, current smokers had almost double the risk of early implant failure (OR 1.9, 95% CI: 1.0–3.6), whereas no increased risk was observed for former smokers (Table 1). A history of severe periodontitis (evidenced by radiographic periodontal bone loss to the apical third of roots) was strongly associated with early implant failure (OR 7.5, 95% CI: 2.4–23). There was evidence for a non‐linear association between maximum bone loss and the risk of implant failure (*p* = 0.003, *p* for non‐linearity = 0.05, Figure 1A). Compared to patients with no periodontal bone loss, patients with a %bone loss/age ratio > 1 had a higher risk of implant failure (OR 3.2, 95% CI: 1.3–8.2). The association between the %bone loss/age ratio and implant failure was linear (*p* = 0.009, *p* for non‐linearity = 0.88, Figure 1B). We also found a linear association between the number of missing teeth and the risk of implant failure (OR 1.4, 95% CI: 1.2–1.7 for an increase by four missing teeth, *p* for non‐linearity = 0.95, Figure 1C). The risk of early implant failure increased with decreasing residual bone height. Compared to sites with 5 mm or more of residual bone, the odds of failure doubled (OR 2.4, 95% CI: 1.4–4.2) for sites with residual bone heights of 3 mm to < 5 mm, and quadrupled (OR 3.8, 95% CI: 2.2–6.8) for sites with residual bone height < 3 mm. There was evidence for a non‐linear association between residual bone height and the risk of implant failure (*p* < 0.0001, *p* for non‐linearity = 0.06, Figure 1D). We did not observe any association between the maximum periodontal bone loss and the residual bone height (Figure 2), suggesting that the increased risks due to a history of severe periodontitis and low residual bone height are independent of each other. Very low primary stability (< 8 Ncm) was associated with a marked increase in the odds of early implant failure (OR 5.8, 95% CI: 1.3–26) compared to implants with insertion torques of 20 Ncm or more. A moderate but statistically significant association was found between lower residual bone height and lower primary stability (*p* for trend = 0.005, Figure 3).

**TABLE 2:** Association between clinical and radiographic parameters and early implant failure after sinus augmentation.

Exposure	Unadjusted model		Multivariable model	
	OR	95% CI	OR	95% CI
Age (10 years)^a^	1.0	(0.8–1.2)	1.0	(0.8–1.3)
Sex^b^				
Female	1.0	Reference	1.0	Reference
Male	1.6	(1.1–2.5)	1.6	(1.1–2.5)
Smoking^c^				
Never	1.0	Reference	1.0	Reference
Former	1.0	(0.6–1.7)	1.0	(0.6–1.8)
Current	1.7	(0.9–3.1)	1.9	(1.0–3.6)
Maximum periodontal bone loss^d^				
No bone loss	1.0	Reference	1.0	Reference
Bone loss coronal third	0.8	(0.4–1.6)	0.8	(0.4–1.5)
Bone loss middle third	1.7	(1.0–3.1)	1.5	(0.8–2.8)
Bone loss apical third	7.2	(2.4–21)	7.5	(2.4–23)
Edentulous	3.1	(1.4–6.8)	2.8	(1.3–6.4)
Ratio bone loss (%)/age (years)^d^				
No bone loss	1.0	Reference	1.0	Reference
0.25–1.0	1.3	(0.8–2.1)	1.2	(0.7–1.9)
> 1.0	3.6	(1.5–8.6)	3.2	(1.3–8.2)
Number of missing teeth (4)^e^	1.4	(1.2–1.5)	1.4	(1.2–1.6)
Residual bone height^f^				
5+ mm	1.0	Reference	1.0	Reference
3 to < 5 mm	2.4	(1.4–4.1)	2.4	(1.4–4.2)
< 3 mm	4.7	(2.7–8.1)	3.8	(2.0– 6.8)
Type of sinus lift^g^				
Transcrestal	1.0	Reference	1.0	Reference
Lateral window	1.5	(0.7–3.1)	0.7	(0.3–1.9)
Membrane perforation^h^	1.8	(1.0, 3.6)	1.3	(0.6, 2.7)
Staging of sinus lift^i^				
One‐stage	1.0	Reference	1.0	Reference
Two‐stage	1.8	(1.1–3.1)	1.7	(0.8–3.7)
Implant length^j^				
13+ mm	1.0	Reference	1.0	Reference
9–12 mm	0.9	(0.4–2.0)	0.7	(0.3–1.7)
≤ 8 mm	5.9	(1.4–24)	2.3	(0.4–13)
Implant diameter^k^				
4.5+ mm	1.0	Reference	1.0	Reference
3.8–4.3 mm	1.3	(0.8–2.1)	1.1	(0.6–2.0)
< 3.8 mm	1.4	(0.7–3.0)	1.2	(0.5–2.9)
Insertion torque^l^				
20+ N cm	1.0	Reference	1.0	Reference
8–19 N cm	0.9	(0.5–1.6)	0.6	(0.3–1.2)
< 8 N cm	5.0	(1.5–17)	5.8	(1.3–26)
Healing mode^m^				
Transmucosal	1.0	Reference	1.0	(Reference)
Submerged	1.0	(0.6–1.6)	0.6	(0.2–1.5)

*Note*: Unadjusted and multivariable adjusted odds ratios (OR) and 95% confidence intervals (CI) are from logistic regression models with case/control status as dependent variable. Parameters in brackets were omitted from the multivariable model based on the 10% change‐in‐estimate criterion.

^a^ Multivariable model adjusted for centre and sex.

^b^ Multivariable model adjusted for centre and age.

^c^ Multivariable model adjusted for centre, age and sex.

^d^ Multivariable model adjusted for centre, age, sex and smoking.

^e^ Multivariable model adjusted for (centre), age, sex, (smoking) and (history of periodontitis).

^f^ Multivariable model adjusted for (centre), age, sex, (smoking), (history of periodontitis) and number of missing teeth.

^g^ Multivariable model adjusted for centre, age, sex, (smoking), history of periodontitis, number of missing teeth and residual bone height.

^h^ Multivariable model adjusted for centre, age, sex, (smoking), history of periodontitis, (number of missing teeth), residual bone height and (type of sinus lift).

^i^ Multivariable model adjusted for centre, age, sex, (smoking), history of periodontitis, number of missing teeth, residual bone height, type of sinus lift and (membrane perforation).

^j^ Multivariable model adjusted for centre, age, sex, (smoking), history of periodontitis, number of missing teeth, residual bone height, (type of sinus lift), (membrane perforation), (staging of sinus lift) and implant diameter.

^k^ Multivariable model adjusted for centre, age, sex, (smoking), history of periodontitis, number of missing teeth, residual bone height, (type of sinus lift), (membrane perforation), (staging of sinus lift) and implant length.

^l^ Multivariable model adjusted for centre, age, sex, (smoking), history of periodontitis, (number of missing teeth), residual bone height, type of sinus lift, (membrane perforation), (staging of sinus lift), (implant length) and (implant diameter).

^m^ Multivariable model adjusted for centre, age, sex, (smoking), history of periodontitis, (number of missing teeth), residual bone height, (type of sinus lift), (membrane perforation), (staging of sinus lift), (implant length), (implant diameter) and insertion torque.

No significant differences in the risk of early failure were found between transcrestal versus lateral window approach, procedures with or without membrane perforations, transmucosal versus submerged healing, one‐stage versus two‐stage procedures or for different implant lengths and diameters (Table 2).

Finally, both former smoking (OR 2.8, 95% CI: 1.1–7.0) and current smoking (OR 3.0, 95% CI: 1.0–8.6) were associated with an increased risk of perforation of the Schneiderian membrane compared to never smoking.

## 4 Discussion

This nested case–control study identified male sex, current cigarette smoking, history of severe periodontitis, number of missing teeth, reduced residual bone height and very low primary stability as risk factors for early implant failure in conjunction with sinus floor augmentation.

Some of these risk factors, such as male sex, smoking and low residual bone height, have been described earlier in smaller studies on external (Testori et al. 2012) or internal SAs (Li et al. 2023). Our findings are also consistent with studies on early implant failure more generally, that is, for implants not necessarily placed in conjunction with sinus floor augmentation. This includes a lack of association of age with implant failure (Sendyk et al. 2017; Srinivasan et al. 2017), as well as an increased risk of males compared to females (Chrcanovic et al. 2015) and smokers compared to non‐smokers (Fan et al. 2024). Smoking is of particular relevance in the context of SA surgery, as it is associated with a higher incidence of intra‐operative perforations of the Schneiderian membrane (Wang et al. 2023). While we could confirm the increased risk of perforations for both former and current smokers in this study, such perforations were not associated with an increased risk of early implant failure, consistent with other studies (Beck‐Broichsitter et al. 2018; Testori et al. 2012). Hence, while membrane perforations are clearly relevant, as they may require the surgeon to modify or even abandon the surgical procedure, they do not explain the effect of smoking on early implant failure.

We found a strong and dose‐dependent association between low residual bone height and the risk of early implant failure. Unsurprisingly, low residual bone height was associated with lower insertion torque, and a very low insertion torque was itself a strong and independent risk factor for early implant failure. However, the association between low residual bone height and insertion torque was moderate in strength and therefore explained only a small part of the association between residual bone height and early implant failure.

Previous studies have shown a higher risk of implant loss in patients with a history of periodontitis compared to periodontally healthy individuals (Chrcanovic et al. 2014). However, evidence specifically for early implant failure is scarce (Noguerol et al. 2006; Olmedo‐Gaya et al. 2016), which is important because a history of periodontitis is now recognised as a strong risk factor for peri‐implantitis (Schwarz et al. 2018), which may eventually lead to implant loss.

In the present study, we found a strong association between a history of severe periodontitis (evidenced by alveolar bone loss into the apical third) and the risk of early implant failure in conjunction with maxillary SA, which—somewhat surprisingly—could not be explained by the lower residual bone height in these patients. Although speculative, a number of factors may contribute to the surprising lack of association between periodontal bone loss and residual bone height, including that pneumatisation of the sinus before and after tooth loss is a much stronger determinant of residual bone height relative to periodontal bone loss and/or that teeth in the relevant region of the maxilla, in particular first molars (which are the most common site in our sample), are lost as a result of caries in some patients before they develop periodontitis. Even though our study is the largest study to date, these results must be cautiously interpreted in the absence of clinical periodontal data and a wide confidence interval. However, subject to confirmation in further studies, this association with the history of severe periodontitis is intriguing and could reflect a common susceptibility to periodontitis that also affects the risk of early implant failure and/or could reflect a causal effect of the periodontal infection itself. While the standard of periodontal care may have varied somewhat in this cohort, it is important to note that even in clinical studies only a minority of patients achieve ‘stable periodontitis’ (Chapple et al. 2018) or reach proposed ‘endpoints of therapy’ (Sanz et al. 2020) following active periodontal therapy (Rattu et al. 2023). Interestingly, studies have suggested a higher risk of peri‐implantitis for patients with residual deep periodontal pockets compared to patients without residual deep pockets after periodontal therapy (Cho‐Yan Lee et al. 2012). However, in the absence of clinical periodontal data, we can only speculate whether a similar association holds for early implant failure; in any case, this would not necessarily mean that more aggressive periodontal treatment would ameliorate the risk (Cho‐Yan Lee et al. 2012).

Our study has several important strengths. It is the largest study to date on the risk of early implant failure after SA. Furthermore, it is based on a large multi‐centre cohort of non‐selected patients, and included a range of experienced surgeons and implant systems, reflecting a broad range of current practice, thereby enhancing the generalisability of our findings. For example, many clinical studies in implantology exclude smokers or at least heavy smokers in an effort to reduce variability. However, smokers lose teeth and request and receive implants, and it is important for clinicians to understand and communicate the associated risks based on evidence.

However, our study has important limitations too. These include the fact that data were collected as part of routine care and clinicians were not calibrated. While this is unlikely to have resulted in misclassification of some parameters (e.g., age and sex), it is more likely to have occurred for others (e.g., insertion torque). Another important limitation is the lack of clinical periodontal data as discussed above. Finally, some exposure categories had scarce data, resulting in imprecise estimates and a lack of power to detect weaker yet clinically meaningful associations.

## 5 Conclusion

In conclusion, we identified male sex, current smoking, low residual bone height, very low insertion torque, a history of severe periodontitis and the number of missing teeth as important risk factors for early implant failure. These findings should be taken into consideration for treatment planning and patient communication.

## Author Contributions

T.D. conceived this work. T.D. and B.B. contributed to study design and data acquisition, and drafted the manuscript. T.D. and M.A. analysed the data. All authors contributed to data interpretation, revised the manuscript critically for important intellectual content and approved the final version.

## Conflicts of Interest

The authors declare no conflicts of interest.

## Data Availability

The data that support the findings of this study are available from the corresponding author upon reasonable request.
